# Estimation of the Content of Selected Active Substances in Primary and Secondary Herbal Brews by UV-VIS and GC-MS Spectroscopic Analyses

**DOI:** 10.1155/2020/8891855

**Published:** 2020-11-12

**Authors:** Jakub Wyrostek, Radosław Kowalski, Urszula Pankiewicz, Ewa Solarska

**Affiliations:** ^1^Department of Analysis and Evaluation of Food Quality, University of Life Sciences in Lublin, 8 Skromna Street, 20-704 Lublin, Poland; ^2^Department of Biotechnology, Microbiology and Human Nutrition, University of Life Sciences, 8 Skromna Street, 20-704 Lublin, Poland

## Abstract

Primary and secondary herbal brews were tested for the content of polyphenolic compounds, flavonoids, and essential oil. The brewing process was carried out at an initial temperature of 95°C and different time parameters (5, 10, 15, and 30 min). A secondary brewing was also carried out to estimate the reuse of the herbal material. The highest content of polyphenol compounds and flavonoids was determined in the primary peppermint brews (4017 mg L^−1^ and 360 mg L^−1^, respectively). The secondary brews were characterised by a lower content of active substances than the primary brews. The study showed that the herbal postbrewing material contained significant levels of essential oil (from 13.04% to 95.65%) and may be an alternative source of volatile bioactive compounds.

## 1. Introduction

Over the recent years, apart from their medicinal and spice functions, herbs have also gained popularity as components of products with health-promoting properties in the form of functional food or diet supplements [1]. It is noteworthy that the current ecological trend is noticeable also in the area of application of herbs in agriculture, in the form of preparations from the group of biopesticides [2]. The assortment of products containing medicinal plants in unprocessed or minimally processed form includes herbal teas which are among the most popular drinks all over the world, due to their exceptional taste and flavour values. They are also one of the oldest forms of herbal medicine and are characterised by a very long tradition in medicine systems all over the world [3]. The raw material provided by medicinal plants is also an interesting object of research, constituting a valuable source of biologically active substances, such as highly water-soluble polyphenols (including flavonoids) and low water-soluble essential oils which, depending on their chemical character, display a broad spectrum of biological activity. Many of those compounds cannot be produced by the human organism, so supplying them with food has also a prophylactic effect, especially by providing protection against free radicals (antioxidant properties) [4].

Plants belonging to the Lamiaceae and Asteraceae families are well known for their high content of polyphenolic compounds, mainly caffeic acid, chicoric acid, cinnamic acid, quercetin, and rosmarinic acid [5]. Peppermint (*Mentha piperita*), chamomile (*Matricaria chamomilla*), and lavender (*Lavandula officinalis*) are the most popular herbs due to their positive effect on the digestive system [6]. The literature also states that caraway and fennel (Apiaceae) are a rich source of polyphenolic compounds such as caffeic acid derivatives [7]. The group of those compounds is characterised by antibacterial, antioxidant, anti-inflammatory, antimicrobial, antiviral, and anticancer activities [8].

An especially interesting and highly diversified chemically group is that of essential oils which were known and used already in ancient times. Nowadays, there can be observed a comeback to the tradition of using essential oils, finding a place for them in various areas. The substances are known for their broad spectrum of biological activity, leading to antibacterial, antimycotic, antivirus, or anti-inflammatory properties of essential oils. The origin of essential oils allows them to be classified among herbal medicines, but due to their high concentration of active substances, their effects are notably stronger than those of liquid herbal brews. The diversity of those substances is so high because they are complex mixtures that may contain over 300 different organic volatile compounds [9].

Herbal brews are characterised by the presence of volatile components that impart a characteristic flavour to, for example, herbal brews. The content of volatile substances has an effect on the health-promoting value of such brews and, hence, the importance of the content of that group of compounds in brews. In addition, the aroma of, for instance, tea brews, affects a number of sensory properties. At present, we have knowledge on the effect of certain flavours on the operation of the brain, on the complex hormonal system, appearance of associations, on emotions, and thus on human behaviour [10].

Active substances are most often acquired from unprocessed plant raw materials, but there is also the interesting possibility of acquisition of biologically active substances from plant waste after industrial processes or even from such waste as may be formed as byproducts during the preparation of meals in our kitchens. Such possibilities of recovery of attractive biologically active substances perfectly fall in with the recently fashionable trend of “green chemistry.” It is worthwhile to manage natural origin material rationally, so that all the components can be suitably utilised, in the production of food, pharmaceuticals, diet supplements, or cosmetics.

Therefore, the objective of the study presented herein was the estimation of concentration of polyphenolic compounds, flavonoids, and essential oils in primary and secondary herbal brews, obtained at various time parameters. In addition, an estimation was made of the possibility of reusing the waste plant material after the performed brewing process, through an analysis of the content of selected biologically active substances in that material. Available literature is very poor in research on the content of essential oil in herbal brews. In addition, the research topics regarding the possibility of using postbrewing plant material to obtain essential oils seem to be a topic that brings an innovative approach to research in this area.

## 2. Materials and Methods

### 2.1. Experimental Material

The plant material used in the experiment was diversified in terms of botanical origin as well as morphology: flowers of lavender *Lavandula angustifolia* Mill. (Flos, Poland), leaves of sage *Salvia officinalis* L. (Flos, Poland), fruits of caraway *Carum carvi* L. (Kawon, Poland), leaves of peppermint *Mentha* × *piperita* L.(Flos, Poland); dried chamomile flowers *Matricaria chamomilla* L. (Flos, Poland), fruits of fennel *Foeniculum capillaceum* Gilib. (Kawon, Poland). Herbs from a single production batch were purchased from a pharmacy. The plant material was standardised through pooling together and fragmentation of a homogeneous fraction, after which the moisture level of each product was measured for conversion to dry matter.

### 2.2. Brewing

**Figure 1:**
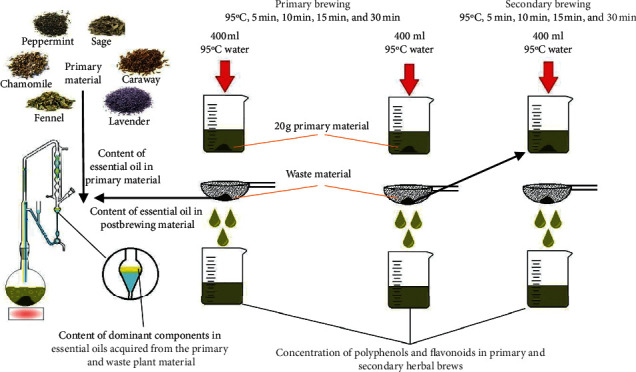
The procedure for sample preparation in the experiment.

Weighed portions of 20 g of herbal material were placed in beakers of 1000 ml in volume and poured over with 400 ml of distilled water with initial temperature of 95°C; then the whole was covered with watch glass. The process of brewing was conducted in various time variants—5 min, 10 min, 15 min, and 30 min (30 min under mechanical stirring). Next, the primary brews were separated from the postbrewing plant material and used for the determination of the content of polyphenolic compounds, flavonoids, and essential oil. In addition, the postbrewing plant material was washed with distilled water at room temperature and subjected again to the process of brewing with distilled water under analogous temperature and time conditions as those described above (secondary brewing). The secondary brews were also analysed for the content of polyphenolic compounds, flavonoids, and essential oil. Each brewing was performed in three replicates and analysed.  Figure 1 illustrates the procedure for samples preparation in the experiment.

### 2.3. Essential Oil Distillation

Distillation of essential oils was conducted in accordance with the procedure described in the Polish Pharmacopoeia VIII. Portions of 20 g of the initial herbal material and the corresponding postbrewing material (after primary brewing, proportions with water 1:20) were subjected to the process of hydrodistillation with Clevenger apparatus (Witeg Labortechnik, Wertheim, Germany) (according to Pharmacopoeia VIII) [11]. From the primary content of oils and their residues in the secondary material, the efficiency of oil extraction and their percentage residue in the secondary material were calculated. The content of essential oil in the plant material was expressed as a volume/weight percentage (% *v*/*w*). All analyses were performed in triplicate.

The distilled oil was collected into tightly closed glass vials, adding anhydrous sodium sulphate for the purpose of drying, and stored at temperature of −20°C until tested and GC analysed.

#### 2.3.1. Essential Oil Concentration in Herbal Brews

The concentration of the essential oil in the obtained herbal brews was calculated on the basis of the difference between the contents of the essential oil in the initial plant material and the plant material after the brewing process, assuming no losses of essential oil in the course of the process of brewing. The concentration of essential oil (EO) in herbal brews was expressed in mg L^−1^ (the density of individual essential oils was taken into account for this calculation): peppermint EO 0.920 g·mL^−1^, chamomile EO 0.917 g·mL^−1^, sage EO 0.914 g·mL^−1^, fennel EO 0.963 g·mL^−1^, lavender EO 0.885 g·mL^−1^, and caraway EO 0.900 g·mL^−1^.

### 2.4. Flavonoid Analysis

Determination of flavonoid content in the tested herbal brews was performed by means of spectrophotometry (*λ* = 425 nm) according to a modified Polish Pharmacopoeia VIII [11] procedure. The results of the flavonoid content were expressed in quercetin equivalents (Sigma-Aldrich, ACS reagent ≥ 98.00%). The results were calculated from the equation of the calibration curve prepared for quercetin standards in the concentration range 1.2–12 mg·L^−1^ (1.2, 1.8, 3, 9, and 12 mg·L^−1^). Each sample, depending on the material of herbal, was diluted appropriately to the range of the standard curve. All analyses were performed in triplicate.

### 2.5. Phenolic Compounds' Analysis

Determinations of phenolic compounds in the tested macerates were made by spectrophotometric means (*λ* = 725 nm) according to a modified Singleton and Rossi method [12]. The results of the phenols content were expressed in gallic acid equivalents (Sigma-Aldrich, ACS reagent ≥ 98.00%). The results were calculated from the equation of the calibration curve prepared for gallic acid standards in the concentration range 10–60 mg·L^−1^ (10, 20, 30, 40, 50, and 60 mg·L^−1^). Each sample, depending on the material of herbal was diluted appropriately to the range of the standard curve. All analyses were performed in triplicate.

### 2.6. Determination of Essential Oil Chemical Composition

The essential oil was diluted in hexane (1:10) and analysed in triplicate.

#### 2.6.1. Gas Chromatography Analysis

*GC/MS*. ITMS Varian 4000 GC-MS/MS (Varian, USA) equipped with a CP-8410 autoinjector and a 30 m × 0.25 mmi·d. VF-5 ms column (Varian, USA), film thickness 0.25 *μ*m, was used; carrier gas He at a rate of 0.5 ml·min^−1^; injector and detector temperature 250°C and 200°C, respectively; split ratio 1:50; and injection volume 0.50 *μ*l. A temperature gradient was applied (50°C for 1 min, then incremented by 4°C min^−1^ to 250°C, and then held at 250°C for 10 min); ionization energy 70 eV; mass range 40–870 Da; and scan time 0.80 s.

*GC/FID.* GC Varian 3800 (Varian, USA) equipped with a CP-8410 autoinjector and a 30 m × 0.25 mm DB-5 column (J&W Scientific, USA), film thickness 0.25 *μ*m, carrier gas helium 0.5 ml·min^−1^, injector and detector FID temperatures 260°C; split ratio 1:100; and injection volume 0.50 *μ*l. A temperature gradient was applied (50°C for 1 minute, then incremented by 4°C min^−1^ to 250°C, 250°C for 10 minutes).

*Qualitative Analysis.* The qualitative analysis was carried out on the basis of MS spectra, which were compared with the spectra of the NIST Library and with data available in the literature [13]. The identity of the compounds was confirmed by their retention indices taken from the literature [13] and our own data for standards (*α*-pinene, limonene, menthone, menthol, linalool, and carvone, Sigma-Aldrich).

*Quantitative Analysis*. The relative percentages of the separated compounds were calculated from integration of the peak areas in the GC chromatograms.

### 2.7. Statistical Analysis

Data were analysed using one-way ANOVA followed by Duncan's test using the SAS statistical system (SAS Version 9.1, SAS Institute, Cary, NC, USA). The significance of all tests was set at *p* < 0.05. Data are shown as mean ± SD. Different letters (a, b, c, etc.) show a significant difference with *p* < 0.05.

## 3. Results and Discussion

### 3.1. Primary Brews

**Figure 2:**
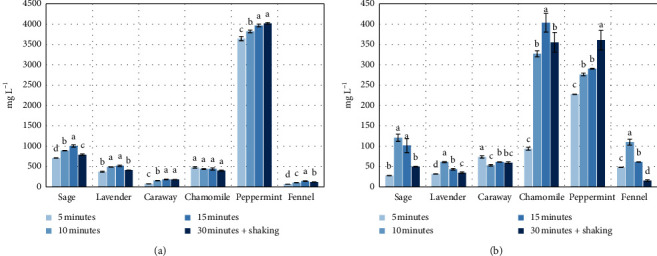
Concentration of polyphenols expressed in gallic acid equivalents (a) and flavonoids expressed in quercetin equivalents (b) in primary herbal brews (mg·L^−1^). Data are shown as mean ± SD. Different letters (a, b, c, etc.) show a significant difference with *p* < 0.05.

The concentration of polyphenols in the primary brews is illustrated in Figure 2(a). With an extension of the time of brewing conducted for the first time from the initial herbal material (95°C, primary brewing), the content of biologically active compounds from the group of polyphenols generally increased for the brews of sage, lavender, fennel, and caraway, attaining the highest concentrations after 15 min (from 141 mg·L^−1^ for fennel to 1006 mg·L^−1^ for sage) (Figure 2(a)). In the case of the mint brews, the highest concentration of polyphenols was found for the brews prepared during 15 and 30 min (3966 mg·L^−1^ and 4018 mg·L^−1^). The chamomile brews did not differ statistically significantly in terms of their polyphenol contents among the particular brews from the plant material prepared in the various time variants (average of 439 mg·L^−1^). The variation of the concentration of polyphenol content in the analysed brews from the particular herbal species results from the content of those substances in the plants, determined by their botanical features. The relation of the maximum concentration of polyphenols in the analysed brews to the kind of the analysed herbs is proportional to their total content converted to gallic acid in the dry matter of the plants as reported in the literature: mint—63 mg·g^−1^ dry matter (DM), sage—24.30 mg·g^−1^ DM [14], chamomile—17.70 mg·g^−1^ DM [15], lavender—16.92 mg·g^−1^ DM [16], fennel—10.17 mg·g^−1^ DM [17], and caraway—3.99 mg·g^−1^ DM [7]. A correlation of the concentration of polyphenols in brews from plants and the duration of the process of brewing was observed also by Dmowski et al. [18] who, as a result of brewing of China black tea conducted for 15 min, obtained higher concentrations of polyphenols in the brew (average of 239.57 mg·L^−1^) compared to shorter brewing times (average of 67.70 mg·L^−1^), which has an impact on the characteristics of the brew in terms of health-promoting properties. A study by Shannon et al. [19] indicates that brewing time of 5 min was optimal for the acquisition of the highest concentrations of polyphenolic compounds in brews from green (557.58 mg·L^−1^) and black (499.19 mg·L^−1^) tea. In a study conducted by Rusinek-Prystupa [20] on the content of active compounds in brews from various species of China tea (black, green, and red), it was found that the content of phenolic acids increased with extension of brewing time (from 1 to 6 min). A similar relationship was observed by McAlpine and Ward [21] who studied the effect of brewing time (1–10 min) of black, green, and herbal teas on the concentration of polyphenols and on the antioxidant activity.

Very high concentrations of flavonoids, at a level approaching ca. 360 mg·L^−1^, were noted for primary brews from mint, which correlates with the highest concentrations of total polyphenols. Analysing the results of flavonoid concentrations in the primary brews, it was observed that in the case of brews from sage, lavender, and fennel, the 10-minute brewing time was optimal for the acquisition of the highest levels of those compounds (120.60 mg·L^−1^ for sage, 60.88 mg·L^−1^ for lavender, and 109.71 mg·L^−1^ for fennel) (Figure 2(b)). In the case of chamomile, the optimum brewing time was 15 min and in that time variant the brew contained 403.50 mg·L^−1^. The 30-minute time of primary brewing of mint was optimal in the case of flavonoids, compared to the shorter times (360.81 mg·L^−1^—30 min, 276.33 mg·L^−1^—5 min, 227.83 mg·L^−1^—10 min, and 290.52 mg·L^−1^—15 min). During the brewing of chamomile, the level of flavonoids migrating to the brew was the highest compared to the other herbs—the maximum of the content of those compounds was obtained in the brew prepared during 15 min—403.53 mg·L^−1^, and only a slightly lower concentration, 355.22 mg·L^−1^, was observed for the 30-minute brewing. The concentration of flavonoids in brews from caraway fruits was characterised by low variation, i.e., from 53.04 mg·L^−1^ (10 min) to 73.59 mg·L^−1^ (5 min). Like in the case of total phenolic compounds, the concentrations of flavonoids in the primary brews from the analysed herbs were varied, which is related with the varied content of those substances in the plants, determined by the botanical features. Adaszyńska-Skwirzyńska and Swarcewicz [22] report that the total content of flavonoids in narrow-leaved lavender varies from 86 mg 100 g^−1^ in the flowers to 618 mg 100 g^−1^ in the leaves. Taking into account the levels of flavonoids content in lavender inflorescences obtained by the authors cited above, and assuming 100% efficiency of brewing, it is possible to achieve flavonoid concentration in brew from inflorescences and leaves (1/20) in the range from about 43 mg·L^−1^ (inflorescences) to 309 mg·L^−1^ (leaves), which corresponds to the concentration in the primary brew from lavender inflorescences in this study (from 31.82 mg·L^−1^ for 5 min to 60.88 mg·L^−1^ for 10 min).

Kazimierczak et al. [23] report that the content of flavonoids in sage leaves is 64.91 mg 100^−1^ g^−1^, which, converted to the brew prepared in this study (1:20), corresponds to the concentration of about 32 mg·L^−1^, and that is in conformance with the concentration in the primary brew from sage leaves in the presented study (from 27.71 mg·L^−1^ for 5 min to 120.60 mg·L^−1^ for 10 min). Frequently, the method of preparation of herbal brews in home conditions during 5 min is not always optimal in terms of achieving the highest concentrations of active substances, valuable in combating numerous diseases, and extension of the brewing time from 5 to 10 minutes can increase their concentration, as in the presented study, even fourfold in the case of sage leaves and dried chamomile flowers.

**Figure 3:**
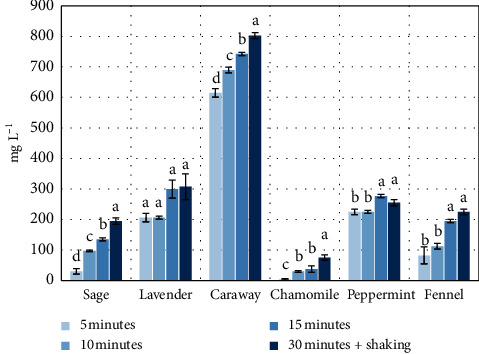
Concentration of essential oils in primary herbal brews (mg·L^−1^). Data are shown as mean ± SD. Different letters (a, b, c, etc.) show a significant difference with *p* < 0.05.

Due to the low solubility of essential oils in water, the efficiency of water extraction and their concentration is relatively low, but the high biological activity of those components allows their health-promoting effects already at low concentrations. As in the case of polyphenolic compounds, the concentration of essential oils in the primary herbal brews was directly proportional to the duration of the brewing process (5–30 min) as shown in Figure 3. In the case of brew from narrow-leaved lavender, the concentration of essential oil varied in the range from 206 mg·L^−1^ to 308 mg·L^−1^, in the brew from leaves of sage—from 30 mg·L^−1^ to 195 mg·L^−1^, from caraway—from 615 mg·L^−1^ to 803 mg·L^−1^, from fennel fruits—from 83 mg·L^−1^ to 225 mg·L^−1^, chamomile—from 4 mg·L^−1^ to 75 mg·L^−1^, and peppermint—from 225 mg·L^−1^ to 255 mg·L^−1^. Tschiggerl and Bucar [24] demonstrated in their study that the concentration of essential oil for fennel brews (15 min) and lavender brews (10 min), as converted to analogous brews, was at the level of 810 mg·L^−1^ and 315 mg·L^−1^, respectively.

**Figure 4:**
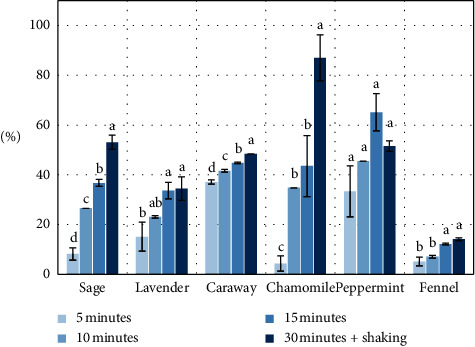
Efficiency of the process of primary brewing for the isolated essential oil (%). Data are shown as mean ± SD. Different letters (a, b, c, etc.) show a significant difference with *p* < 0.05.

Figure 4 illustrates the efficiency of the process of primary brewing for the isolated essential oil. The data indicate that the highest level of extraction efficiency was a characteristic of chamomile (86.96%) and then of peppermint (65.15%), sage (53.06%), caraway (48.42%), and lavender (34.45%), and the lower efficiency was noted in the case of fennel (14.08%). As water extraction liberates essential oil only to a small extent, in the case of repeat use of postbrewing material, one should consider the application of a lipophilic solvent, for example, a vegetable oil, that would be medium allowing to conduct a process of extraction with a high degree of efficiency. Such oil extracts could be used for the production of diet supplements.

### 3.2. Secondary Brews

Wang et al. [25] found that each successive brewing (secondary brewing) significantly reduced the content of catechins and phenolic compounds in the brews obtained. Secondary brewing leads to an effective isolation of biologically active substances, which allows repeated use of herbal residues from the process of preparation of, for example, brews.

**Figure 5:**
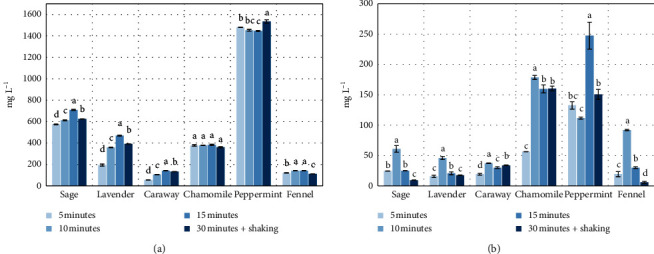
Concentration of polyphenols expressed in gallic acid equivalents (a) and flavonoids expressed in quercetin equivalents (b) in secondary herbal brews (after primary brewing) (mg L^−1^). Data are shown as mean ± SD. Different letters (a, b, c, etc.) show a significant difference with *p* < 0.05.

Analysing the concentration of polyphenols in the secondary brews (Figure 5(a)), generally lower concentrations of those substances in the brews were observed (for 15 min brews: sage 1006.24 mg·L^−1^—primary brew, 709.23 mg·L^−1^—secondary brew; lavender 519.20 mg·L^−1^—primary brew, 470.01 mg·L^−1^—secondary brew; caraway 182.96 mg·L^−1^—primary brew, 142.19 mg·L^−1^—secondary brew; chamomile 440.84 mg·L^−1^—primary brew, 383.54 mg·L^−1^—secondary brew; and mint 3966.48 mg·L^−1^—primary brew, 1455.42 mg·L^−1^—secondary brew). In the case of fennel fruits, the concentration of polyphenols after secondary infusion was higher than in the case of primary maceration (for 5 min brews: 65.24 mg·L^−1^—primary brews, 121.16 mg·L^−1^—secondary brews; for 10 min brews: 101.61 mg·L^−1^—primary brews, 142.28 mg·L^−1^—secondary brews). Fruits of fennel are characterised by relatively high hardness, compared to the other experimental materials, and hence, it can be supposed that as a result of primary maceration, their anatomical structures undergo a certain “loosening,” due to which the secondary brewing is more effective. In the case of the concentration of flavonoids in secondary brews, also lower concentrations were found compared to the primary brews (sage 120.60 mg·L^−1^—primary brews, 60.88 mg·L^−1^secondary brews; lavender 60.88 mg·L^−1^—primary brews, 45.78 mg·L^−1^—secondary brews; caraway 73.59 mg·L^−1^—primary brews, 37.37 mg·L^−1^—secondary brews; chamomile 403.53 mg·L^−1^—primary brews, 178.78 mg·L^−1^—secondary brews; fennel 109.71 mg·L^−1^—primary brews, 92.03 mg·L^−1^—secondary brews; and mint 360.81 mg·L^−1^—primary brews, 247.52 mg·L^−1^—secondary brews).

**Figure 6:**
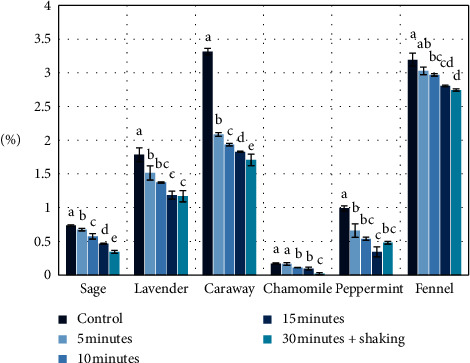
Content of essential oil in the postbrewing (after primary brewing) plant material (% *v*/*w*). Control—content of essential oil in the primary material. Data are shown as mean ± SD. Different letters (a, b, c, etc.) show a significant difference with *p* < 0.05.

Analysis of the content of essential oil in the postbrewing plant material indicates that it can be a valuable raw material for the acquisition of volatile components (Figure 6). The brewing process caused the liberation of essential oil from the secretory structure only to a small extent because essential oils have poor solubility in water. The highest content of essential oil was noted for fennel fruits and caraway, at 3.20% *v*/*w* and 3.32% *v*/*w*, respectively, whereas in the postbrewing material from fennel and caraway, the following levels of essential oil content were obtained: for fennel from 2.75% *v*/*w* (30 min) to 3.03% *v*/*w* (5 min) and for caraway from 1.71% *v*/*w* (30 min) to 2.09% *v*/*w* (5 min.). The content of essential oil in flowers of narrow-leaved lavender was 1.79% *v*/*w*, while after the process of brewing, the postbrewing (waste) plant material contained from 1.17% *v*/*w* (30 min) to 1.52% *v*/*w* (5 min) of essential oil. In the case of leaves of sage, the levels of essential oil were as follows: in the initial raw material—0.74% *v*/*w* and in the postbrewing material—from 0.35% *v*/*w* (30 min) to 0.68% *v*/*w* (5 min). The content of essential oil in peppermint was 0.99%, while after the process of brewing the plant material contained from 0.35% *v*/*w* (15 min) to 0.66% *v*/*w* (5 min) of essential oil. The lowest content of essential oil was noted in the case of chamomile—0.17% *v*/*w*, while in the postbrewing chamomile material, the content assayed was from 0.02% *v*/*w* (30 min) to 0.17% *v*/*w* (5 min). Analysis of the content of the essential oil in the postbrewing material showed significant percentage residue of these biologically active substances calculated from the difference in extraction efficiency and content in the primary material in the range of 46.94%–91.84% (salvia), 65.55%–84.87% (lavender), 51.58%–62.90% (caraway), 13.04%–95.65% (chamomile), 34.85%–66.67% (peppermint), and 85.92%–94.84% (fennel) compared to the primary material, depending on the brewing time (Figures 2–4).

### 3.3. Essential Oil Composition

Quantitative differences in the content of essential oil in the initial plant materials are determined primarily by the botanical features. In addition, each of the analysed essential oils was characterised by a different chemical composition which determines the health-promoting properties of products containing that volatile component.

Table 1 presents the percentage contents of the main components of essential oil obtained from the initial plant material and from the postbrewing material (secondary or waste material). The dominant components of lavender oil included linalool, at a share of up to 28.41%, and linalool acetate, with a share of up to 19.03%. Adaszyńska et al. [26] also found that the main component of lavender oil was linalool (8.63–23.88%), and among other components, they enumerate linalyl anthranilate (1.58–12.78%), *α*-terpineol (4.00–7.90%), terpinen-4-ol (5.53–9.73%), lavandulol (3.38–4.64%), geranyl acetate (2.37–10.61%), and caryophyllene oxide (2.12–5.14%). Observing the changes in the contents of the main components of essential oils from the waste raw materials in relation to the prior process of brewing, in some cases one can note certain trends (Table 1). In the case of lavender oil, it was observed that the level of linalool acetate decreased from 19.03% (primary material) to 14.88% (secondary material after brewing conducted for 30 min). Lavender oil is characterised by antibacterial, antimycotic, smooth muscle relaxing, sedative, and antidepressant effects and is effective against burns and insect bites and stings. In addition, it is used in aromatherapy and as a flavouring agent for soaps [27].

In the presented study, the dominant components in the composition of sage oil were camphor (16.57%), *cis*-thujone (16.31%-control), viridiflorol (15.44%), and *trans*-thujone (8.37%). Zawiślak [28] reports that the main components of sage oil were *α*-thujone (up to 21.51%), camphor (up to 18.08%), and 1,8-cineole (up to 18.04%). In the case of sage oil, also a trend was observed, where the contents of *cis*-thujone and camphor decreased, respectively, from 16.31% (primary material) to 13.92% (secondary material after brewing conducted for 30 min) and from 16.57% (primary material) to 11.53% (secondary material after brewing conducted for 30 min). This may indicate a better water solubility of these two main components of the essential oil compared to the others, resulting in a lower content in the waste material. In the case of viridiflorol in sage oil, an opposite trend was observed, i.e., in oil acquired from the primary, unprocessed material, a content of 10.10% of that component was noted, while for the secondary, postbrewing plant materials, sage oils had a higher levels of viridiflorol: from 12.02% (secondary sample after 5 min brewing) to 15.44% (secondary sample after 30 min brewing). Sage oil and its preparations have antiperspirant properties, display anti-inflammatory effects, and inhibit microbial growth; they are used in cases of infections of mucous membranes of the throat and mouth (inflammation of the mouth, gums, and throat) [29].

Dominant components in caraway oil were carvone (up to 53.05%) and limonene (up to 47.88%), which is also supported by the studies by Venskutonis et al. [30] where in oils acquired from a caraway collection limonene contents up to 55.40% and carvone up to 44.40%. In caraway oil, the content of carvone increased from 46.80% (primary raw material) to 53.05% (secondary sample after 30 min brewing). Caraway oil has antispastic effects, prevents flatulence, and improves the peristalsis of the digestive tract, and it is an antiseptic agent (against *Escherichia coli* and *Staphylococcus aureus)* [31], as well as antiasthmatic [32].

Analysing the essential oil from fennel fruits, one can note that the dominant component is *trans*-anethole, the content of which in the primary sample was 76.67%. The second compound in terms of content in fennel soil was fenchone (14.26%). In the waste postbrewing material, the content of those compounds decreased with the extension of the process duration. A slight increase in the content of *trans*-anethole was noted at maceration time extension from 15 to 30 min with stirring. As reported by [33], oil from fennel fruits has a dominant content of anethole (65%), fenchone (22%), and also limonene (3.50%), *α*-pinene (3.00%). Fennel oil displays mainly carminative effects, antibacterial, lactogogic, cholagogic, antiparasitic, and antimycotic.

The basic components of oil from dried chamomile flowers in the experiment included *α*-bisabolol oxide A (50.89%), *α*-bisabolol oxide B (10.01%), and *α*-bisabolone oxide A (6.80%). As reported by Szőke et al. [34], the contents of the individual components in chamomile oil depend primarily on the chemotype of the plant from which it is extracted. The first chemotype is characterised by a higher content of bisabolol oxide, and the second contains more *α*-bisabolol. The dominant components of chamomile oil are *α*-bisabolol (up to 60%), *α*-bisabolol oxide A (up to 60%), *α*-bisabolol oxide B (up to 50%), chamazulene (i.p. to 25%), and *α*-bisabolone oxide A (up to 12%) [35]. Chamomile oil is used mainly as a component of complex preparations used in disorders of the alimentary tract.

The dominant component in the composition of peppermint oil was menthol, the content of which in oil acquired from the initial primary material was 41.72%. With the extension of the process duration of brewing, the percentage concentration of that compound decreased slightly, down to 38.76% in oil from plant material after 30-minute brewing. Other important compounds in the composition of peppermint oil were menthone and menthyl acetate, with levels of up to 15.57% and 13.88%, respectively. Góra et al. [35] report the following levels of the dominant components: menthol up to 80%, menthone up to 45%, and methyl acetate up to 30%. Peppermint essential oil is commonly used in folk medicine in diseases of the respiratory system as an apophlegmatic preparation and antispastic and antibacterial in the digestive and vascular systems [36]; it alleviates the symptoms of the irritable bowel syndrome in people [37].

The individual components of essential oils are characterised by various levels of stability in water systems, especially in the case of application of a high temperature (water solubility, volatility, chemical reactivity, etc.), which results from differences at the molecular level. Therefore, brewing process conducted at an initial temperature of 95°C may result in changes in the content of the individual components, which, in the examples presented here, can be observed in the form of decreasing or increasing trend lines for the percentage contents of volatile components in the distilled oils.

## 4. Conclusions

The use of the calculation method to determine the concentration of essential oils in infusions made it possible to evaluate the infusions in terms of the content of volatile substances. Essential oils are a very important group of active substances that determine the health-promoting properties of herbal infusions.

The results of the study indicate that the duration of herbal brewing has a direct effect on the concentration of the extracted active compounds. The content of flavonoids was the highest in brews prepared during 5–10 minutes brewing, with the exception of mint and chamomile for which the highest concentration of those compounds was found in brews prepared during 30 and 15 minutes, respectively.

It was demonstrated that the optimum time of water brewing of the herbs, conducted with initial temperature of 95°C, was 15 minutes and that time allowed to achieve the maximum concentration of polyphenolic compounds in the prepared brews, whereas the maximum concentration of essential oils in the brews was obtained for brewing time of 30 minutes.

The significant levels of biologically active substances in the secondary brews indicate that the postbrewing plant material can be an alternative raw material for the acquisition of various extracts or pure biologically active components. The study showed that the herbal postbrewing material contained significant levels of essential oil (from 13.04% to 95.65%) and may be an alternative source of volatile bioactive compounds.

## Figures and Tables

**Table 1:** Comparison of the content of dominant components in essential oils acquired from the primary and waste plant material.

Component	RI	RI_Lit_	Most fragment ions with R_Int_ (%)	Primary material	Content (%)^*∗*^			
					Waste material after the maceration process			
					5 min	10 min	15 min	30 min
Peppermint								
Menthone	1149	1148	112 (100%), 139 (79%), 81 (64%)	14.02^c^ ± 0.33	13.82^c^±0.16	14.92^b^ ± 0.13	15.30^a^ ± 0.11	15.57^a^ ± 0.17
Menthol	1168	1167	71 (100%), 41 (92%), 81 (88%)	41.72^a^ ± 0.57	41.12^a^ ± 0.18	39.37^a^ ± 0.43	39.15^b^ ± 0.21	38.76^b^ ± 0.27
Menthyl acetate	1295	1294	43 (100%), 95 (82%), 81 (65%)	10.65^c^ ± 0.28	13.88^b^ ± 0.09	12.61^b^ ± 0.10	12.58^b^ ± 0.13	12.35^a^ ± 0.09

Chamomile								
*α*-Bisabolol oxide B	1650	1656	43 (100), 105 (42), 143 (38)	10.01^c^ ± 0.50	10.96^b^ ± 0.05	11.45^b^ ± 0.24	11.06^b^ ± 0.31	13.43^a^ ± 0.26
*α*-Bisabolone oxide A	1677	1684	43 (100), 93 (74), 67 (44)	6.80^d^ ± 0.15	7.59^c^ ± 0.14	8.39^b^ ± 0.11	7.73^c^ ± 0.21	10.77^a^ ± 0.29
*α*-Bisabolol oxide A	1740	1748	238 (2), 43 (100), 143 (58), 93 (29)	50.89^cd^ ± 1.43	52.82^b^ ± 0.58	49.68^d^ ± 0.45	54.83^a^ ± 0.29	52.19^bc^ ± 0.59

Fennel								
Fenchone	1079	1083	152 (1), 81 (100%), 69 (52%), 41 (31%)	14.26^b^ ± 0.04	14.16^c^ ± 0.09	14.74^a^ ± 0.04	14.10^c^ ± 0.01	13.94^d^ ± 0.01
*trans*-Anethole	1280	1282	148 (100%), 147 (44%), 117 (29%)	76.69^a^ ± 0.05	76.17^b^ ± 0.19	76.15^b^ ± 0.27	75.77^c^ ± 0.07	75.85^c^ ± 0.04

Lavender								
Linalool	1094	1095	154 (1), 71 (100%), 43 (87), 93 (69%)	26.46^d^ ± 0.10	28.41^a^ ± 0.21	27.57^b^ ± 0.17	26.96^c^ ± 0.07	26.54^d^ ± 0.19
Linalool acetate	1255	1254	93 (100%), 43 (90%), 41 (56%)	19.03^a^ ± 0.40	16.29^b^ ± 0.11	15.84^c^ ± 0.13	15.35^d^ ± 0.03	14.88^e^ ± 0.14

Sage								
*cis*-Thujone	1098	1101	152 (4%), 81 (100%), 41 (85%), 67 (60%)	16.31^a^ ± 0.27	15.87^ab^ ± 0.27	15.53^b^ ± 0.25	14.69^c^ ± 0.36	13.92^d^ ± 0.27
*trans*-Thujone	1109	1112	152 (2%), 81 (100%), 41 (98%), 67 (72%)	8.36^a^ ± 0.05	8.33^a^ ± 0.08	8.37^a^ ± 0.05	8.00^b^ ± 0.18	7.86^b^ ± 0.14
Camphor	1141	1141	152 (4%), 95 (100%), 81 (54%), 108 (46%)	16.57^a^ ± 0.35	14.44^b^ ± 0.15	13.81^c^ ± 0.18	12.78^d^ ± 0.28	11.53^e^ ± 0.23
Viridiflorol	1589	1592	222 (1%), 43 (100%), 109 (55%), 69 (43%)	10.10^e^ ± 0.16	12.02^d^ ± 0.11	13.97^c^ ± 0.04	14.70^b^ ± 0.04	15.44^a^ ± 0.13

Caraway								
Limonene	1021	1024	136 (17%), 68 (100%), 93 (60%), 67 (43%)	47.88^a^ ± 0.21	47.61^a^ ± 0.23	40.22^d^ ± 0.20	47.07^b^ ± 0.14	43.17^c^ ± 0.16
Carvone	1236	1239	150 (9%), 82 (100%), 54 (49%), 93 (34%)	46.80^e^ ± 0.15	47.20^d^ ± 0.13	48.17^c^ ± 0.11	48.89^b^ ± 0.11	53.05^a^ ± 0.19

RI: retention indices, RI_Lit_: retention indices taken from the literature [13], and R_Int_ (%): relative intensities. ^*∗*^Data are shown as mean ± SD. Different letters (a, b, c, etc.) in the line show a significant difference with*p* < 0.05.

## Data Availability

The data used to support the findings of this study are included within the article.

## Supplementary Materials

Supplementary Materials Figure Is: GC-MS chromatogram with dominant components of sage essential oil. Figure IIs: GC-MS chromatogram with dominant components of lavender essential oil. Figure IIIs: GC-MS chromatogram with dominant components of caraway essential oil. Figure IVs: GC-MS chromatogram with dominant components of chamomile essential oil. Figure Vs: GC-MS chromatogram with dominant components of peppermint essential oil. Figure VIs: GC-MS chromatogram with dominant components of fennel essential oil.
