# Supplementary material for: Estimation of the Content of Selected Active Substances in Primary and Secondary Herbal Brews by UV-VIS and GC-MS Spectroscopic Analyses
Source: J Anal Methods Chem. 2020 Nov 12;2020:8891855. doi: 10.1155/2020/8891855 (PMC7676977; doi:10.1155/2020/8891855)
Supplement: Supplementary Materials — Figure Is: GC-MS chromatogram with dominant components of sage essential oil. Figure IIs: GC-MS chromatogram with dominant components of lavender essential oil. Figure IIIs: GC-MS chromatogram with dominant components of caraway essential oil. Figure IVs: GC-MS chromatogram with dominant components of chamomile essential oil. Figure Vs: GC-MS chromatogram with dominant components of peppermint essential oil. Figure VIs: GC-MS chromatogram with dominant components of fennel essential oil. [file 8891855.f1.docx]

**Supplementary material**

**
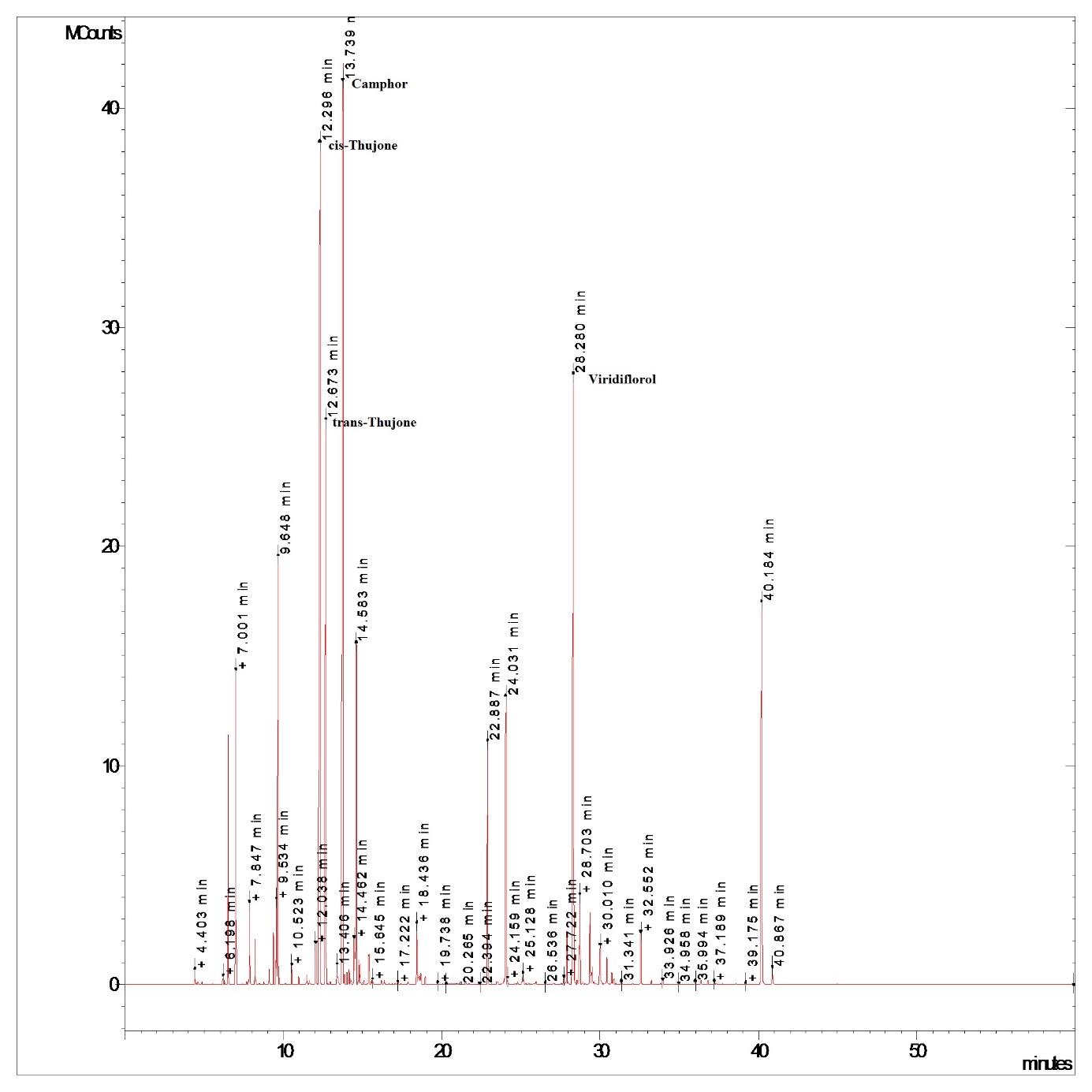
**

**Fig. Is.**

GC-MS chromatogram with dominant components of sage essential oil


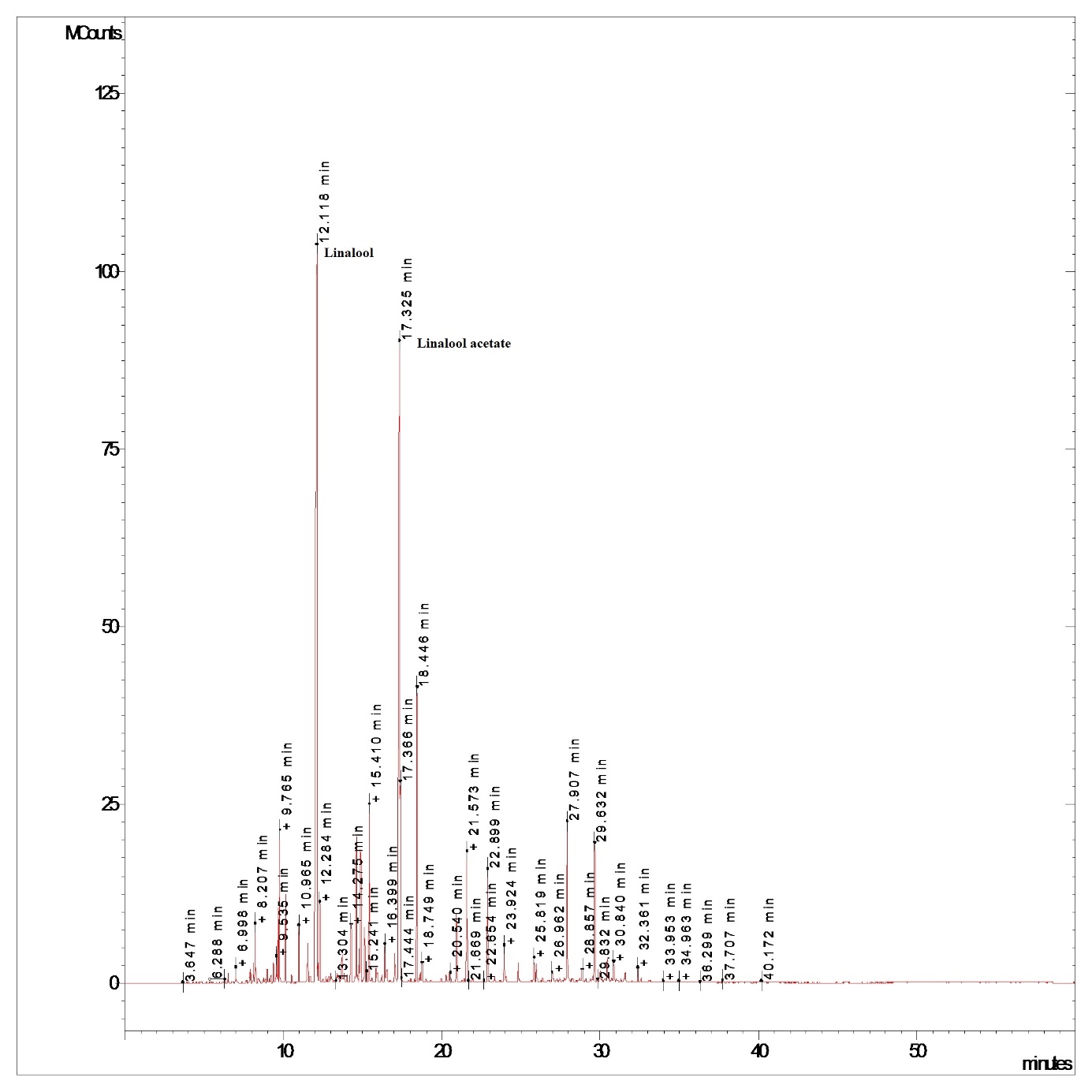


**Fig. IIs.**

GC-MS chromatogram with dominant components of lavender essential oil


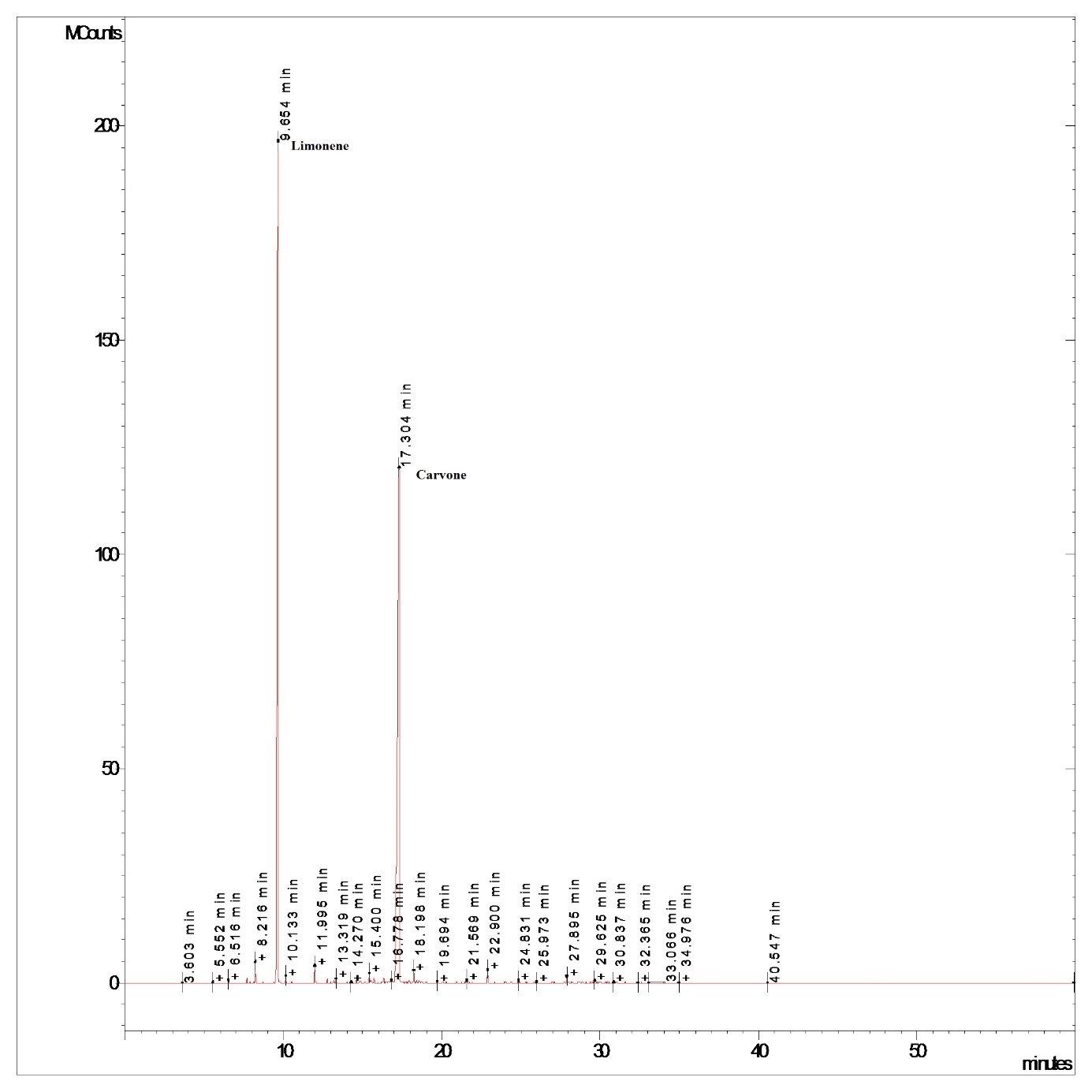


**Fig. IIIs.**

GC-MS chromatogram with dominant components of caraway essential oil


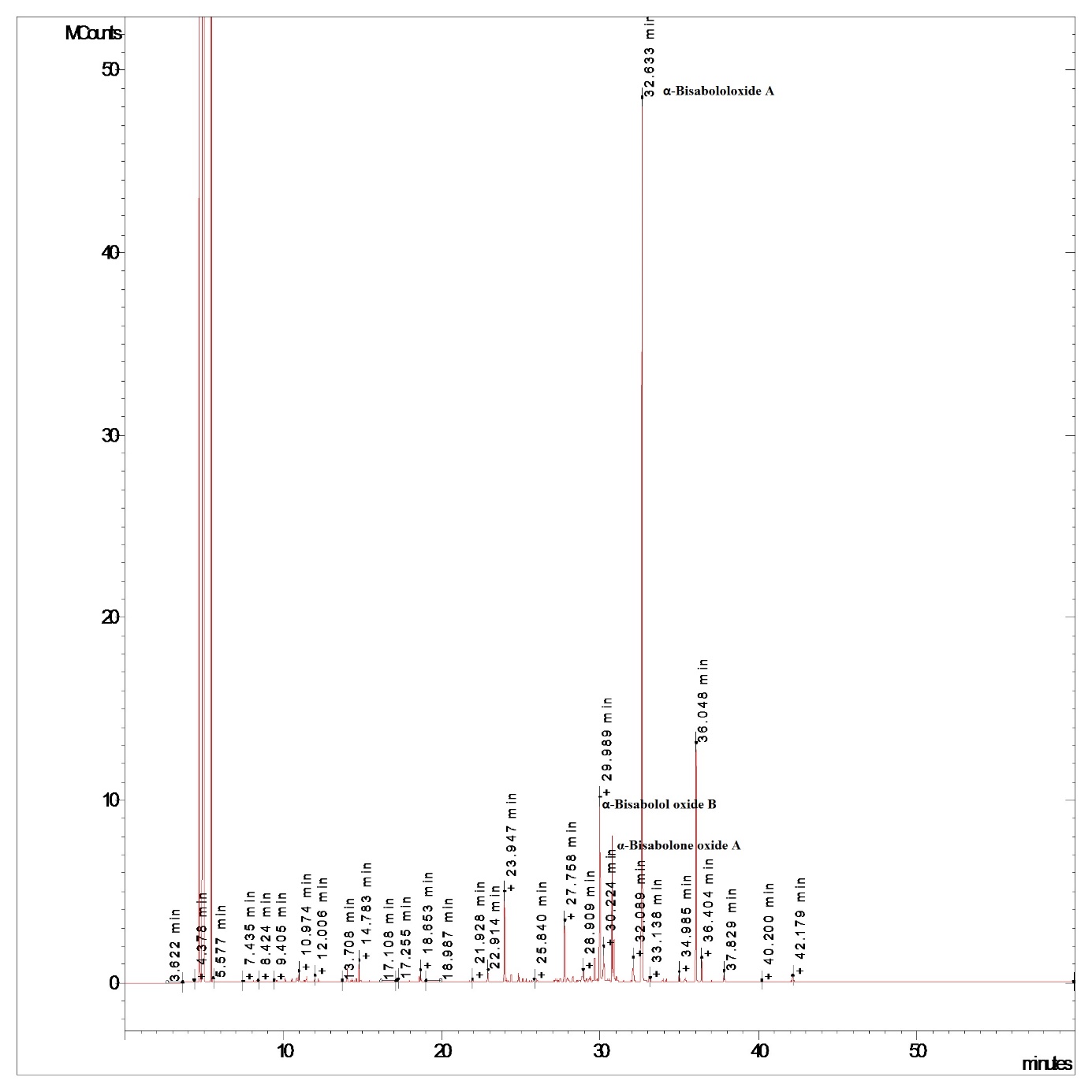


**Fig. IVs.**

GC-MS chromatogram with dominant components of camomile essential oil


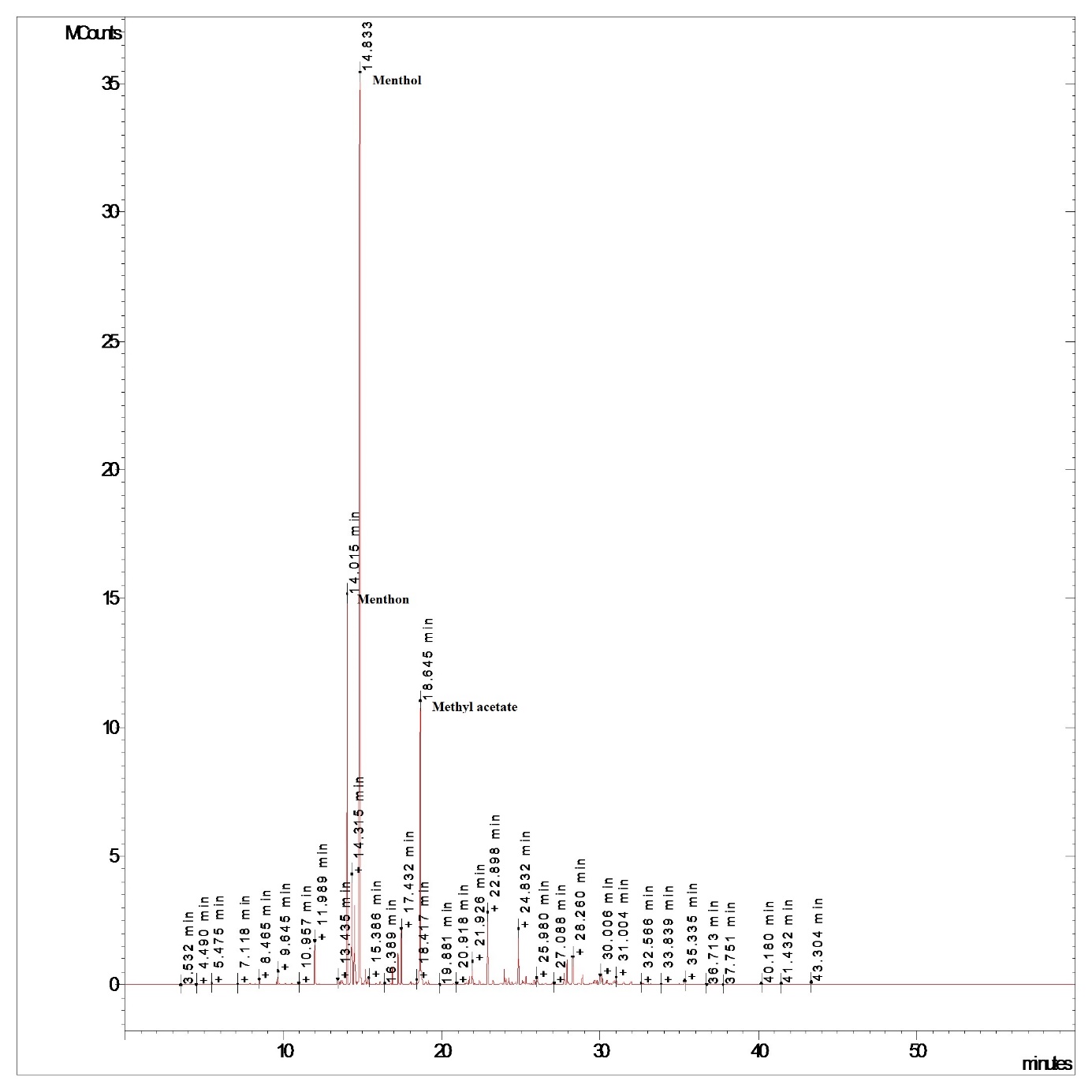


**Fig. Vs.**

GC-MS chromatogram with dominant components of peppermint essential oil

**
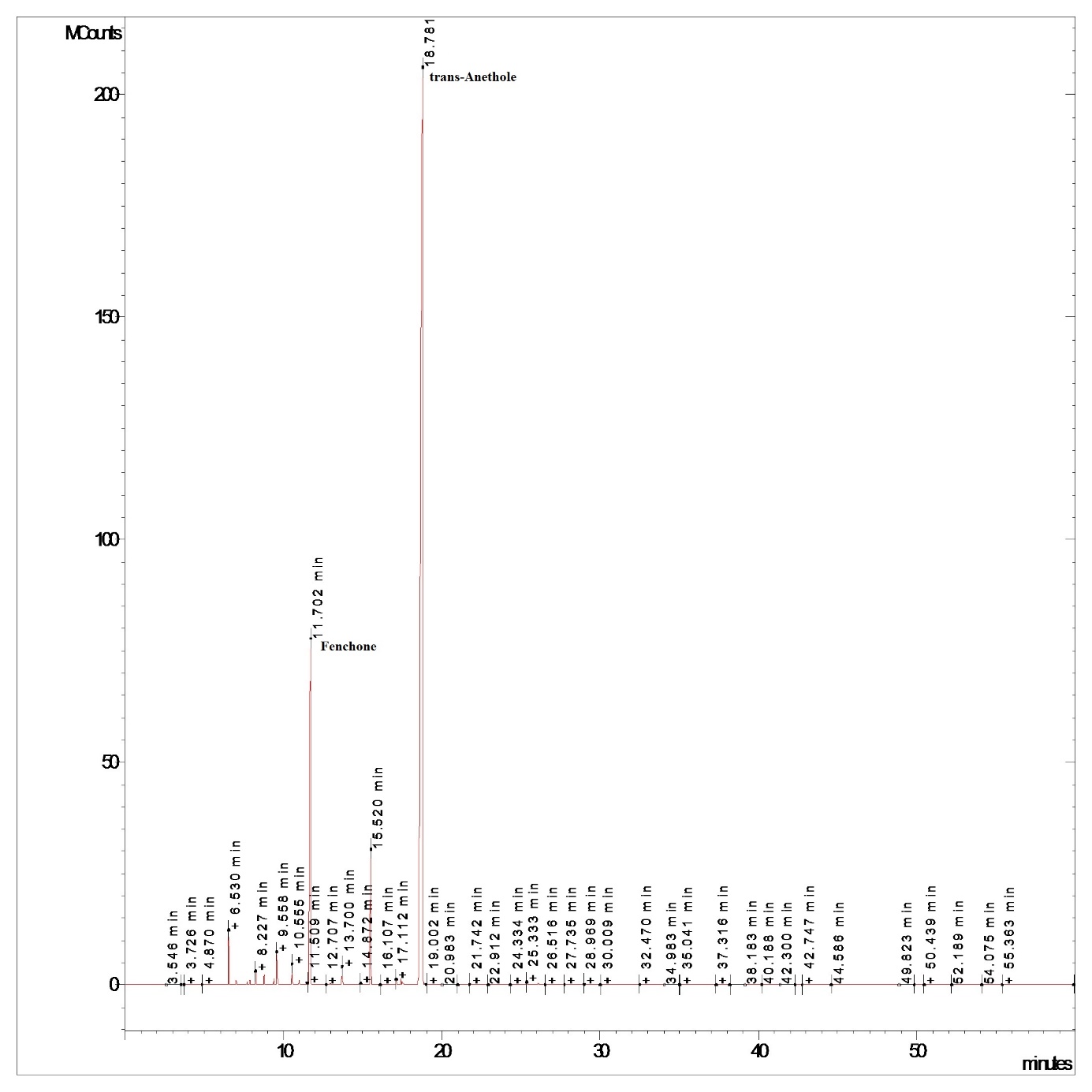
**

**Fig. VIs.**

GC-MS chromatogram with dominant components of fennel essential oil
